# Nonrandom Composition of Flower Colors in a Plant Community: Mutually Different Co-Flowering Natives and Disturbance by Aliens

**DOI:** 10.1371/journal.pone.0143443

**Published:** 2015-12-09

**Authors:** Takashi T. Makino, Jun Yokoyama

**Affiliations:** 1 Department of Biology, Faculty of Science, Yamagata University, 1-4-12 Kojirakawa, Yamagata, Japan; 2 Institute of Regional Innovation, Yamagata University, Yujiri 19–5, Kanakame, Kaminoyama, Yamagata, Japan; Central China Normal University, CHINA

## Abstract

When pollinators use flower color to locate food sources, a distinct color can serve as a reproductive barrier against co-flowering species. This anti-interference function of flower color may result in a community assembly of plant species displaying mutually different flower colors. However, such color dispersion is not ubiquitous, suggesting a variable selection across communities and existence of some opposing factors. We conducted a 30-week study in a plant community and measured the floral reflectances of 244 species. The reflectances were evaluated in insect color spaces (bees, swallowtails, and flies), and the dispersion was compared with random expectations. We found that co-existing colors were overdispersed for each analyzed pollinator type, and this overdispersion was statistically significant for bees. Furthermore, we showed that exclusion of 32 aliens from the analysis significantly increased the color dispersion of native flowers in every color space. This result indicated that aliens disturbed a native plant–pollinator network via similarly colored flowers. Our results demonstrate the masking effects of aliens in the detection of color dispersion of native flowers and that variations in pollinator vision yield different outcomes. Our results also support the hypothesis that co-flowering species are one of the drivers of color diversification and affect the community assembly.

## Introduction

The myriad colors of animal-pollinated flowers are one of the most fascinating examples of biodiversity in the world [1–3]. One of the keys to understanding the driving forces underlying this phenomenon is their ecological function, which has been mainly explored in the context of interactions with pollinating animals, i.e., flower color as an advertisement to attract pollinators [4,5]. For example, flower colors are finely tuned to pollinator vision [6–8] and preference [9–12] in order to repeatedly attract pollinators, which is a requirement for successful pollination.

Apart from mimicry species [13–17], the flower color of a species is expected to be different from that of co-flowering species [18–20]. That is, a distinct color helps in attracting pollinators exclusively to a particular species, thereby transferring pollen reliably to conspecifics, saving pollen otherwise wasted on heterospecifics, and preventing stigma clogging with heterospecific pollen grains [21–23]. Various pollinators use color stimuli to locate food items, often consistently selecting a certain color over different colors as a result of innate or learned preferences [9,24–30] while ignoring subtle differences between nearly identical colors [31–35]. Thus, selection for different color as a reproductive barrier may yield a community of co-flowering species with mutually different flower colors. Indeed, such color overdispersion has been detected in a subtribe of Andean Solanaceae [36], but not in *Pedicularis* [20] or *Oxalis* [35].

Detection of overdispersed flower colors is rare in community studies covering flowering species across a range of taxa. Gumbert et al. [18] investigated five plant communities for eight months and evaluated the colors of 168 species in the bee color space. They compared the observed dispersion of flower colors with random expectations but did not find community-level overdispersion. In a different approach, using 44 species, McEwen and Vamosi [19] found significant overdispersion in only one of five communities. The selection for different colors may not have been strong enough for overdispersion to occur ubiquitously. To develop a general overview, however, we need more examples, and we should consider the factors opposing color dispersion. Here we suggest two such factors.

First, alien species may disturb the color composition of native species. Given that an original composition is established through long-term interactions among native species, aliens without such a history might not fit into the native composition. The expected composition, in which co-existing colors are dissimilar, might not emerge unless we exclude aliens from the analysis. This effect can be very noticeable when the alien and native flower colors are similar. Indeed, although the reflectances are unknown, there are some examples where flowers of native and alien species have a similar colors, at least to a human eye [37–39]. However, it is also possible that successful aliens are those that have found niches unoccupied by similarly colored natives; such color vacancy may help invasion. In such cases, aliens can reinforce color dispersion.

Second, the interpretation of color composition might depend on the vision of the pollinators. While bees have trichromatic vision [40], certain swallowtails possess tetrachromatic vision, which covers a wider range of wavelengths in comparison with the eyes of bees that lack a red-type receptor [34]. Furthermore, flies have a categorical perception of wavelength, where any color falls into one of four color types [31]. Considering such differences can improve our understanding of flower colors. For example, some bird-pollinated flowers exhibit colors that are more easily detectable by birds than by bees [7]. In Ohashi et al [41], variations in pollinator vision yield different outcomes in the evaluation of plant species that appears to change flower color. Thus, evaluating flower color with the vision of various pollinators may provide us with different aspects of flower color composition.

To explore these factors, we conducted a 30-week study on co-flowering species in a rural area of Japan. We identified 212 native and 32 alien species of presumably animal-pollinated plants. Their floral reflectances were evaluated in insect color spaces, and the color differences between co-flowering species were computed both for the real community and for a hypothetical community without aliens. We then performed randomization tests to find out whether the exclusion of aliens significantly affected the color differences among co-flowering species. We also evaluated whether flower colors were more divergent (or convergent) than they would be by chance. All these analyses were performed in the color spaces of different pollinators: bees, swallowtails, houseflies, and droneflies. Taking into account the composition of flower colors seen through the eyes of different pollinators, we discussed the effect of aliens on the color composition of native flowers and the effect of co-flowering species as drivers of flower-color diversity.

## Materials and Methods

### Determination of co-flowering species

To examine as many flowering species as possible, we defined six census trails, each of which passed through various habitats (dry and humid meadows, a forest floor of deciduous trees, ridges between rice fields, etc.) in a rural area of Kaminoyama, Yamagata, Japan (S1 Fig). These trails were located outside Zao Quasi-National Park and no specific permissions were required for most of these trails; for the rice fields, we have obtained permission from the owner. The total length of the trails was approximately 2.6 km, with an average inter-trail distance of approximately 0.4 km. We walked through the census trails once a week from April 16 (early spring) to November 5, 2014 (late fall), to identify the composition of co-flowering species. Plant species were categorized as native or alien according to the invasive species list provided by National Institute for Environmental Studies (http://www.nies.go.jp/biodiversity/invasive/resources/listen_toc.html). A species of *Nymphaea*, which is not on the list but is a wild-growing cultivar, was labeled as alien. As they were difficult to identify in the field, two *Oxalis* species (*O*. *stricta* and *O*. *dillenii*) were treated as a single native species. Our 30-week investigation revealed the flowering phenology of 244 animal-pollinated species (33 Orders, 76 Families) consisting of 212 natives and 32 aliens (S2 Fig, S1 Table). No endangered or protected species were involved. Because various habitats were intermingled in the study site, the pollinators could encounter all co-flowering species. We, therefore, treated all the data as representing a single plant community that shared the same pollinator fauna.

### Spectral measurements

We measured the diffuse spectral reflectance of flowers with a spectrometer (BRC115P-U, B&W Tec, Inc., Delaware, USA), relative to a white reference (SRR, B&W Tec, Inc., Delaware, USA), under a deuterium/tungsten light source (BDS100, B&W Tec, Inc., Delaware, USA) in a range of 300–700 nm. When a flower had multiple colors, we targeted its most “showy” part (the largest area). For example, for daisy species, we selected a ray floret (its outer region was selected when the ray floret had two colors); for a legume flower, we selected its banner petal. Because we focused on the most attractive parts, what we targeted was not necessarily a petal: for example, we selected the basal bract of *Houttuynia cordata*, as it has no showy petals. For species with changing flower color, such as *Rubus parvifolius*, younger flowers were sampled. For dioecious species with differently sized male and female flowers, such as *Akebia*, we selected larger flowers. For species without size difference, we pooled the reflectance data. We sampled 10 flowers for each species (with a few exceptions), and the mean reflectance curve was used for analysis. The locations and sample sizes are summarized in S1 Table.

### Evaluation of floral reflectance in insect color space

We evaluated floral reflectance in the color spaces of three pollinator types: bees, butterflies, and flies. We chose these species because they are major pollinators whose chromatic representations are well studied. Birds were not considered because they seldom visited the flowers in our study site (pers. obs.). The procedure we used was similar to that adopted by Ohashi et al. [41]. They used the representative spectral sensitivities of honeybees (*Apis mellifera*), swallowtails (*Papilio xuthus*), and houseflies (*Musca domestica*). We added droneflies (*Eristalis tenax*) as the second representative of flies (also in Suzuki and Ohashi [42]). The spectral sensitivity of the photoreceptors was taken at a 5-nm step size according to Chittka and Kevan [43], Koshitaka et al. [34], Arnold et al. [44], and Lunau and Wacht [45]. With an assumption that the receptors were adapted to the green foliage background, we calculated the relative amount of light absorbed by a receptor (i.e., quantum catch) as follows:

$$P=\frac{\int_{300}^{700}\;S\left(\lambda \right)I\left(\lambda \right)D_{65}\left(\lambda \right)d\lambda }{\int_{300}^{700}\;S\left(\lambda \right)I_{B}\left(\lambda \right)D_{65}\left(\lambda \right)d\lambda },$$

where *I* is the spectral reflectance of a flower; *I*_B_ is the spectral reflectance of the green foliage [43]; *S* is the spectral sensitivity of the receptor; *D*_65_ is the standard illuminant of daylight [43]; and *dλ* is the wavelength step size (5 nm in this study). *P* was transformed into the input to the brain (*E*) assuming that its maximum was set to 1. The process is described by

$$E=\frac{P}{P+1}$$

We then plotted the values of *E* in the color space of each pollinator type and estimated the color difference perceived by the flower visitors (hereafter called “color distance”).

For bees, with their trichromatic vision, we selected the color hexagon model [40]. We calculated the color loci (*x*- and *y*-coordinates), and the color distance between species *i* and *j* (*d*_*ij*_) as follows:

$$x=\frac{\sqrt{3}}{2}\times \;\left(E_{\text{G}}-E_{\text{U}}\right)$$

$$y=\;E_{\text{B}}-0.5\;\times \;\left(E_{\text{G}}-E_{\text{U}}\right)$$

$$d_{ij}=\;\sqrt{{\left(x_{i}-x_{j}\right)}^{2}+{\left(y_{i}-y_{j}\right)}^{2}},$$

where *E*_*G*_, *E*_*U*_, and *E*_*B*_ are the inputs from green, UV, and blue receptors, respectively.

For swallowtails, with their tetrachromatic vision [34], we adopted the approach of Ollerton et al. [7], with which they evaluated the color distance perceived by birds. The *x*-, *y*-, and *z*-coordinates and the color distance between species *i* and *j* (*d*_*ij*_) were calculated as follows:

$$x=E_{\text{U}}-\frac{\left(E_{\text{B}}+E_{\text{G}}+E_{\text{R}}\right)}{3}$$

$$y=E_{\text{B}}-\frac{\left(E_{\text{U}}+E_{\text{G}}+E_{\text{R}}\right)}{3}$$

$$x=E_{\text{G}}-\frac{\left(E_{\text{U}}+E_{\text{B}}+E_{\text{R}}\right)}{3}$$

$$d_{ij}=\;\sqrt{{\left(x_{i}-x_{j}\right)}^{2}+{\left(y_{i}-y_{j}\right)}^{2}+{\left(z_{i}-z_{j}\right)}^{2}}$$

where *E*_G_, *E*_U_, *E*_B_, and *E*_R_ are the inputs from green, UV, blue, and red receptors, respectively.

For houseflies and droneflies, we chose the model proposed by Troje [31], which predicted the wavelength discriminability of blowflies (*Lucilia* sp.). Their chromatic representation is based on the relative excitations of the two p-type and two y-type receptors: UV vs. blue (p-type) and violet vs. green (y-type). The color perceived by flies is determined by the receptor of each pair with the greater excitation. Thus, flower color falls in one of the four categories (p+y+, p+y–, p–y+, and p–y–) according to the following equations:

$$p=\;E_{\text{U}}-E_{\text{B}}$$

$$y=\;E_{\text{V}}-E_{\text{G}},$$

where *E*_U_, *E*_B_, *E*_V_, and *E*_G_ are the inputs from UV, blue, violet, and green receptors, respectively. Because colors in the same category are considered to be indistinguishable to flies [31], we defined the color distance between species *i* and *j* (*d*_*ij*_) as 0 when both colors were in the same category and as 1 when they were in different categories.

### Calculation of dissimilarity index

Because the composition of co-flowering species varies with time, a species can be relatively conspicuous or cryptic depending on when it flowers. In other words, each species has its own suitable flowering period to be dissimilar (or similar) to the others. To evaluate whether a species produced flowers when it became relatively conspicuous or cryptic, we used a dissimilarity index (*D*). The index was calculated in the following manner: First, as the criterion, we computed the expected mean color distance (*E*_*i*_) for each species as follows:

$$E_{i}=\frac{\sum_{j}^{n}\;d_{ij}}{n-1},$$

where *d*_*ij*_ is the color distance between species *i* and *j* (*d*_*ii*_ is 0) and *n* is the total number of observed species. In other words, *E*_*i*_ is the mean color distance from species *i* to all other species. Second, we computed the observed mean color distance (*O*_*i*_) for each species using the following equation:

$$O_{i}=\frac{\sum_{k}\;d_{ik}}{n_{i}},$$

where *d*_*ik*_ is the color distance between species *i* and its co-flowering species *k* and *n*_*i*_ is the total number of co-flowering species for species *i*. For example, if species *x* was seen flowering on two observation days during which species *a*, *b*, and *c* were also flowering on at least one day, *O*_*x*_ was calculated as (*d*_*xa*_ + *d*_*xb*_ + *d*_*xc*_)/3. Finally, the *D* for species *i* was defined by

$$D_{i}=O_{i}-E_{i},$$

The index exceeds 0 if a flower color differs more from those of co-flowering species than expected (*O* > *E*), and it is less than 0 if a flower color is more similar to the others than expected (*O* < *E*). For each pollinator type, we calculated *D* for every species for the observed community including alien species and for the hypothetical community excluding alien species (*n* = 244 and 212, respectively). We also calculated *D*_*i*_/*E*_*i*_ as the effect size.

### Test against null composition (randomization test 1)

Because *D*s are not independent of each other, applying a standard statistical method (e.g., *t*-test for *D* assuming a zero mean as a null hypothesis) is inappropriate. Therefore, we conducted a randomization test to evaluate the likelihood of the observed mean *D*. The null hypothesis is that flower colors are randomly assigned to plant species irrespective of the color of co-flowering species. To generate the null data, we shuffled the 244 observed flower colors among the 244 plant species without changing their phenology and then calculated the mean *D*. This was repeated 10,000 times to create a null distribution of mean *D*. Based on the distribution, we determined the likelihood (*P*-value) of the observed mean *D* in a two-sided manner. We conducted the test for each pollinator type and also applied the same procedure to the data set excluding aliens.

### Test for the effect of alien species (randomization test 2)

If the flower colors of the aliens and of the co-flowers are similar, the exclusion of aliens raises the mean *D* and *vice versa*. To estimate the likelihood of the observed change, we compared it with the null distribution. We removed 32 randomly selected species from the complete data and calculated the change in the mean *D* (the number of removed species simulates the number of alien species). This process was repeated 10,000 times, after which we computed the *P*-value in a two-sided manner. The test was performed for each pollinator type.

These multiple tests using the same data might increase the risk of type I errors. Therefore, we adjusted the *P*-values in a table-wide manner using the method of Benjamini and Hochberg [46].

### Comparison of *D*s in natives and aliens (randomization test 3)

To examine the differences between *D*s for natives and aliens, we conducted another randomization test. We shuffled the label of origin (i.e., native or alien) among the 244 plant species and calculated the difference between the mean *D* of pseudo-natives and that of pseudo-aliens. This was repeated 10,000 times to create a null distribution of difference; based on this, we determined the *P*-value of the observed difference in a two-sided manner. The test was performed for each pollinator type.

### Comparison of flowering duration in natives and aliens

During the field research, we noticed an apparent difference between the flowering durations of native and alien plants. We defined the flowering duration of a species as the number of weeks in which we saw the species flowering. To examine the differences between the flowering durations of native and alien species, we applied a generalized linear model with a log link function and Poisson error distribution. Origin (native or alien) was treated as a fixed factor. We used Poisson error distribution because the flowering duration values in this study were count data.

## Results

**Fig 1:**
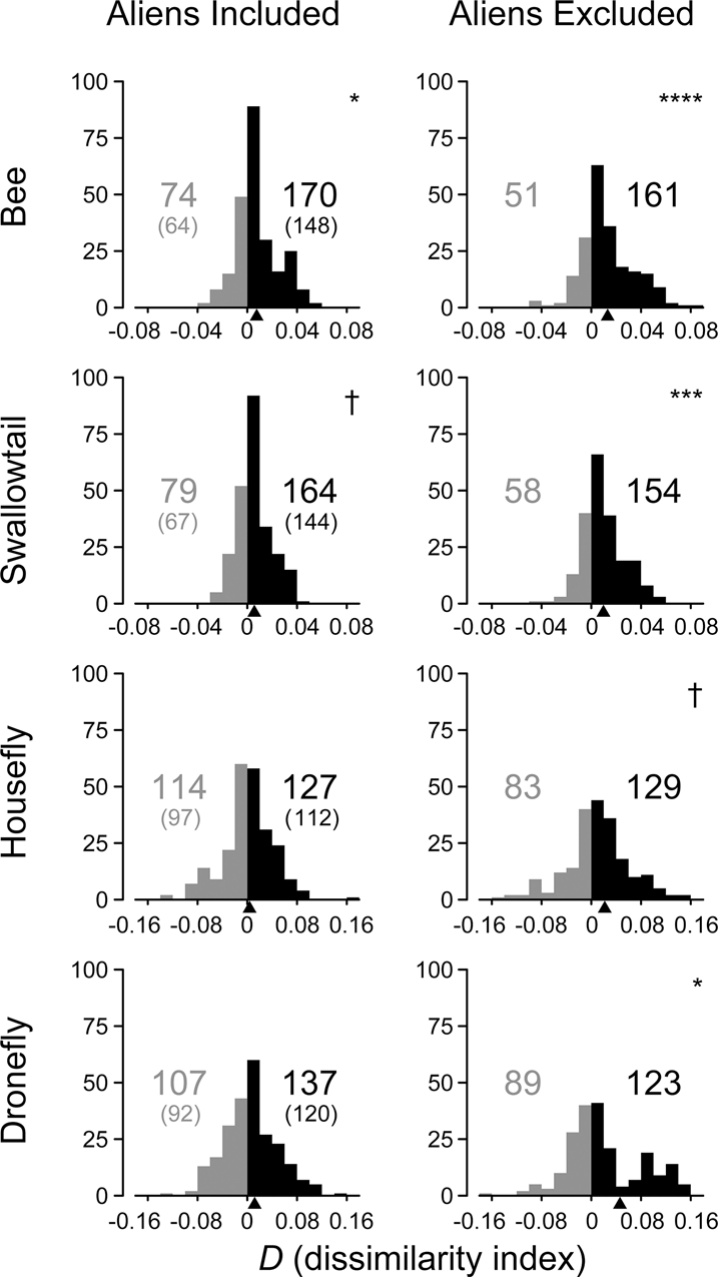
Histograms of the dissimilarity index *D* of flower colors evaluated in the insect color spaces of four pollinator types. The left column shows the results for both native and alien species (*n* = 244); the right column shows the results excluding aliens (*n* = 212). The number of species with negative *D* (left) and positive *D* (right) is shown in each panel. The number of native species is shown in brackets. The triangle below the *x*-axis indicates the mean *D*. The symbols show the adjusted *P* values of randomization tests for the mean *D* (†, *P* < 0.10; *, *P* < 0.05; ***, *P* < 0.001; ****, *P* < 0.0001).

Co-flowering species tended to exhibit mutually different flower colors, as indicated by the positive means of *D*s for all pollinators (Table 1). The mean *D* was significantly higher than random expectation only for bees in the community including aliens. In the hypothetical community without aliens, the mean *D* was significantly higher for bees, swallowtails, and droneflies (Fig 1). Exclusion of aliens significantly increased the mean *D* for all pollinators (Table 1), with simultaneous increases in the number of natives with dissimilar colors (Fig 1). Briefly, flower colors were dissimilar among co-flowering natives, but aliens blurred the composition of natives with mutually different colors.

**Table 1:** Mean *D* and *D*/*E* evaluated by each pollinator’s color space and the results of randomization tests for mean *D*.

Pollinator	Aliens included (*n* = 244)		Aliens excluded (*n* = 212)		Change in mean *D* (*D*/*E*) with exclusion	
	mean *D* (*D*/*E*)	adjusted *P* ^a^	mean *D* (*D*/*E*)	adjusted *P* ^a^		adjusted *P* ^b^
Bee	**0.0076** (0.0365)	0.0240	**0.0131** (0.0637)	< 0.0001	**+0.0056** (+0.0272)	< 0.0001
Swallowtail	0.0057 (0.0270)	0.0533	**0.0097** (0.0468)	0.0030	**+0.0041** (+0.0198)	0.0008
Housefly	0.0019 (0.0033)	0.7202	0.0108 (0.0200)	0.0914	**+0.0090** (+0.0167)	0.0468
Dronefly	0.0059 (0.0223)	0.4728	**0.0230** (0.0720)	0.0180	**+0.0171** (+0.0497)	0.0130

Significant *D*s are indicated in bold.

^a^ Adjusted *P* values of randomization test 1.

^b^ Adjusted *P* values of randomization test 2.

**Fig 2:**
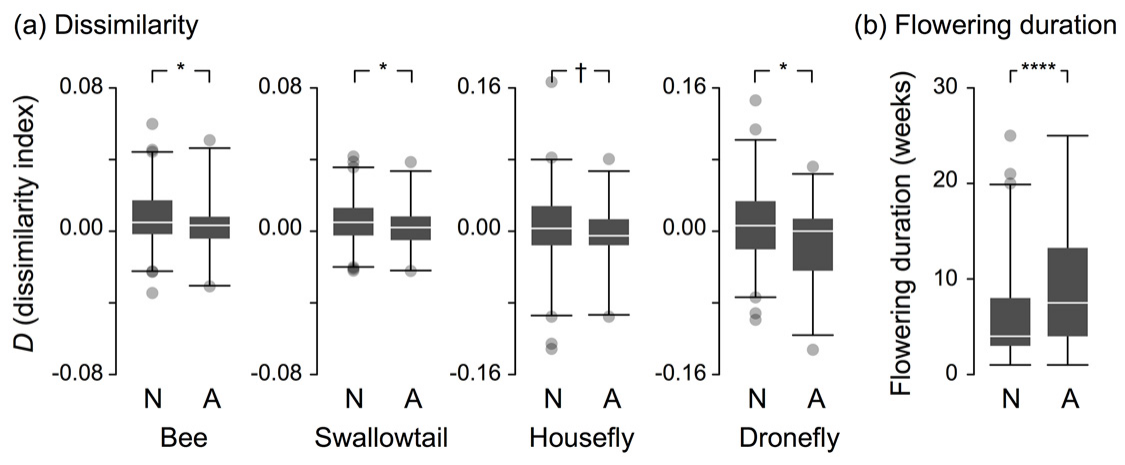
Box-and-whisker plot of (a) the dissimilarity and (b) the flowering duration of natives (N) and aliens (A). The band in each box, the bottom and the top of the box, and the ends of the whiskers represent the median, the 25th and 75th percentiles, and 1st and 99th percentiles, respectively. Small circles are outliers. *n* = 212 and 32 for natives and aliens, respectively. Symbols show (**a**) the adjusted *P* values of randomization test 3 and (**b**) the *P* value of GLM (†, *P* < 0.10; *, *P* < 0.05; ****, *P* < 0.0001).

In the observed community, aliens had lower *D* than natives for all pollinator types, and the difference was significant for bees, swallowtails, and droneflies (Fig 2A). The lower *D* meant that aliens were less likely to avoid similar colors than natives. Aliens flowered over longer periods than natives (Fig 2B).

## Discussion

We detected a significant overdispersion of flower colors (Table 1, Fig 1), in agreement with the findings of McEwen and Vamosi [19]. This result supports the hypothesis that interactions among co-flowering species drive the diversification of flower colors and affect the process of species assembly from a pool of available species across various taxa [47].

This is the first study to demonstrate the disrupting effect of aliens on nonrandom color composition (Table 1, Fig 1), with several implications for the species invasion discussed later. We also found variation in color composition perception among different pollinator types: flower colors appeared significantly dissimilar to bees and marginally dissimilar to swallowtails but not to flies. The variation remained after the exclusion of aliens although the exclusion significantly enhanced the color dispersion for every pollinator (Table 1, Fig 1). These findings suggest that the aliens and unsuitable pollinator vision can mask the important aspects of a color composition and that variations in pollinator vision yield different outcomes; these factors should receive particular attention in the future studies.

### Generality of overdispersed flower colors in a plant community

Although we detected an overdispersion of flower colors, such overdispersion has not been very noticeable in previous studies. Gumbert et al. [18] identified 168 species from five sites and found overdispersed flower colors in two sites. However, this overdispersion was found only for rare species and none of the common species. McEwen and Vamosi [19] identified 44 species in five sites and observed significant overdispersion only in one site. Similarly, variable results also have been obtained in the studies of close relatives. While Muchhala et al. [36] detected color overdispersion in Andean Solanaceae, Eaton et al. [20] found no overdispersion in *Pedicularis* and de Jager et al. [35] actually reported a color convergence in *Oxalis*.

The sporadically detected overdispersion of flower colors might be partly explained by the effects of aliens and unsuitable color evaluations, which we focused on in this study. However, the inconsistent results suggest that the benefit of distinct floral colors varies across species and communities. While floral color divergence is expected to reduce reproductive interference among co-flowering species [21–23], color convergence can also benefit plants. For example, some unrewarding species attract pollinators by mimicking the flower colors of rewarding species [13,16,17], and even rewarding species can benefit from the increased attractiveness achieved by color convergence [15]. This benefit appears important for rare species that lack the ability to appeal to pollinators by themselves [18]. In addition, the benefit of color convergence outweighs the cost of reproductive interference if a plant species has another way to avoid such interference. One example is placing the pollen onto different body parts of the pollinators [48,49]. The color convergence of *Oxalis* flowers may be pertinent because their pollinators are likely to use some cues other than color to discriminate between the similarly colored species [35]. It is also possible that flower colors are unimportant and therefore neutral to selection. Eaton et al. [20] detected neither significant color divergence nor convergence in *Pedicularis*. However, they found overdispersion in other floral traits, sufficient to avoid interference. The overall tendency of flower color dispersion in a plant community probably depends on the proportion of plant species that benefit from the color difference. Therefore, the overdispersion sporadically detected across different communities may provide hints to the determinants of the tradeoff between color divergence and convergence. However, we did not find anything in common among the “hot spots” where color overdispersion was detected. This result suggests that more examples are needed.

We should mention a possible mechanism for the color overdispersion detected in this study. To calculate the dissimilarity index (*D*), we computed the mean color distance from a given species to all other species as the expected distance (*E*) and the same distance for only those species that co-flower (*O*) and then subtracted *E* from *O*. This method allows a species with a longer flowering duration to have larger effects than a species with a shorter flowering duration, because the color of a species with a longer flowering duration affects *D*s of more co-flowering species. For example, if there is one species with a very unusual color and long flowering duration, it will not increase *E* much for any given species but will increase *O* (and *D*) of many co-flowering species. The overdispersion in this study can be explained by a possible tendency of rare colored plants to have a longer flowering duration. To test this possibility, we analyzed the relationship between flowering duration and *E* (a higher *E* indicates more unusual color) and found very weak correlations (S2 Table). The positive but small coefficients (0.07 on average) suggest that the contribution of the focal mechanism was minor, at least in this study.

### Effect of aliens on natives via color disturbance

Although the colors of native flowers showed significant overdispersion for most pollinator types, the alien flowers offset it to some extent (Fig 1). Decreased flower color dispersion in a community suggests that aliens reduce the chromatic barrier against interspecific movements of the pollinators, thereby interfering with the reproductive success of the natives.

### Process of species assembly showing overdispersed flower colors

The process of species assembly is of major interest in community ecology [47,50,51]. Armbruster et al. [48] suggested that communities are assembled in a manner that reduces interference among the co-existing species. In agreement with this notion, we detected overdispersed flower colors. This result indicated that the interference mediated by flower colors played an important role in the construction of the focal community, particularly the original community before alien invasion. However, it is difficult to decide whether the overdispersion among natives resulted from competitive exclusion between similarly colored flowers and/or character displacement in flower color or flowering phenology. A comparison of intraspecific variations in floral traits in multiple communities could establish the relative likelihood of competitive exclusion and character displacement.

Our results have some implications for the process of alien invasions. As shown in Fig 2A, aliens exhibit slightly but significantly lower *D* than natives for most pollinator types. This result indicates that aliens have a weaker tendency to avoid similarly colored flowers than native plants. In addition, aliens had significantly longer flowering duration than natives (Fig 2B). A longer flowering duration lets a plant species interact with more co-flowering species and therefore increases the likelihood of encountering similarly colored heterospecifics. Aliens with a lower *D* and longer flowering duration might be less sensitive to reproductive interference.

It should be noted that our list of native species is not necessarily identical to the original species assemblage. Some unrecorded natives might have been made extinct by human activities or displaced by aliens with similar ecological niches. More research on communities with the historical records of species assemblages may clarify this issue.

### Differences among pollinator types and relationship with the pollination syndrome

Although the statistical significance varied among the pollinator types (Table 1, Fig 1), the mean *D* was positive for every pollinator type. This result indicated that there were no substantial differences between the trends of color composition; flower colors were basically dispersed for every pollinator type. The insignificant dispersion for flies might have been caused by their lower color resolution. In contrast to the vision models that allow bees and swallowtails to recognize many colors, we applied a model of categorical color perception to flies, following Troje [31] and other studies [42,44,52]. In the categorical system, any spectral reflectance falls into one of the four color types; flies are therefore less sensitive to the overdispersion of flower colors than bees and swallowtails. It is also possible that the color composition is primarily adjusted to the dominant pollinators. However, this seems unlikely because swallowtails are far less abundant than flies. Such adjustment may occur in case of droneflies but not in case of houseflies; there were some differences between these two groups in the hypothetical community without aliens (Fig 1). However, there is no available data on the abundance of these two types of flies.

The competition for pollinators appears to be more intense among plants depending on the same pollinators than among those depending on different pollinators. Thus, color dispersion may become more apparent when we separately analyze the plant species according to the pollination syndrome [11]. For example, if we analyze only bee-pollinated flowers using a bee vision model, we would expect to detect a stronger overdispersion of flower colors. In addition, both mean *D* and effect size (*D*/*E*) would be higher. In the present analysis of both natives and aliens (Table 1), the mean *D* for bees was 0.0076, which corresponds to an approximately 3.3% improvement in the recognition of color difference by *Bombus terrestris* [33]. These values seem small but are likely to increase when the pollination modes are taken into account. This approach is worth trying in future studies although it is difficult to identify the pollination modes for every species in a whole plant community (244 species in this study). At present, even the relative abundance of pollinator types is unknown for this site. However, based on a personal observation during the field research, there exist at least some major pollinators (bees, flies, butterflies, beetles, and moths) including honeybees, swallowtails, houseflies, and droneflies, which is similar to the typical pollinator fauna in the temperate areas of Japan. On the other hand, we have never seen birds and bats visiting flowers in our site.

We also emphasize our detection of overdispersion even without considering the pollination syndrome. This may reflect that a plant species can benefit from flower color difference even when the co-flowering species depend on different pollinators. If the pollinators recognize any flower-like colors as the cues for food sources, similarly colored flowers will distract the desirable pollinators even when the flowers depend on different pollinators. It is also possible that there is no clear pollination syndrome prevailing in our community; the plant species may not be specialized for a particular pollinator type but may share generalized pollinators [53,54]. Identification of flower visitors and a hierarchical analysis of color dispersion should answer the questions related to the pollination syndrome.

### Underestimation due to phylogenetic constraints

Because close relatives sharing similar genetic backgrounds are likely to produce flowers in similar colors ([55–58], but see [19,58]), our analysis, which does not incorporate such phylogenetic constraints, might have underestimated color dispersion. Thus, the estimates in Table 1 might be conservative. To examine this possibility, we grouped similarly colored species in the same genus (see S3 Table for the method and the list of grouped species) and found several expected changes (S4 Table, S3 Fig). The insignificant mean *D* for swallowtails in the analysis including aliens became significant. The mean *D* for houseflies, which was insignificant even in the analysis without aliens, became significant after this grouping. Although the phylogenetic constraint does not always decrease color dispersion [19], we should keep this in mind in cases of apparent low color dispersion.

## Supporting Information

S1 Fig Map of the six census trails. The trails are shown in thick lines.
(PDF)

S2 Fig Flowering phenology of 244 species for 30 weeks. The horizontal line indicates weeks in which a species was seen flowering. Alien species are shown in red. The number on the left side of a line is a species ID (S1 Table).
(PDF)

S3 Fig Histograms of the dissimilarity index *D* of flower colors after grouping similarly colored species in the same genus. The left column shows the results for both native and alien species (n = 244); the right column shows the results excluding aliens (n = 212). In this analysis, similarly colored species in the same genus were grouped and treated as a single species (see S3 Table for the method and the list of grouped species). The number of species with negative *D* (left) and positive *D* (right) is shown in each panel. Unlike Fig 1, the numbers of natives are not shown on the left panels because some groups contain both natives and aliens. The triangle below the x-axis indicates the mean D. The symbols show the results of randomization tests for the mean D (*, *P* < 0.05; **, *P* < 0.01).
(PDF)

S1 Table Species list. (PDF)

S2 Table Kendall's *τ* for correlations between *E* and flowering duration. (PDF)

S3 Table List of similarly colored species in the same genus for each pollinator vision. (PDF)

S4 Table Results of randomization tests after grouping similarly colored species in the same genus. (PDF)

S1 Spreadsheet Spectral reflectances of 244 species. (XLSX)

S2 Spreadsheet Flowering phenology of 244 species. (XLSX)

S3 Spreadsheet Flowering duration of 244 species. (XLSX)

S4 Spreadsheet Dissimilarity index shown in Fig 1, Fig 2, and S3 Fig. (XLSX)
