# Supplementary figures and images for: Nonrandom Composition of Flower Colors in a Plant Community: Mutually Different Co-Flowering Natives and Disturbance by Aliens
Source: PLoS One. 2015 Dec 9;10(12):e0143443. doi: 10.1371/journal.pone.0143443 (PMC4674055; doi:10.1371/journal.pone.0143443)

**S1 Fig.**

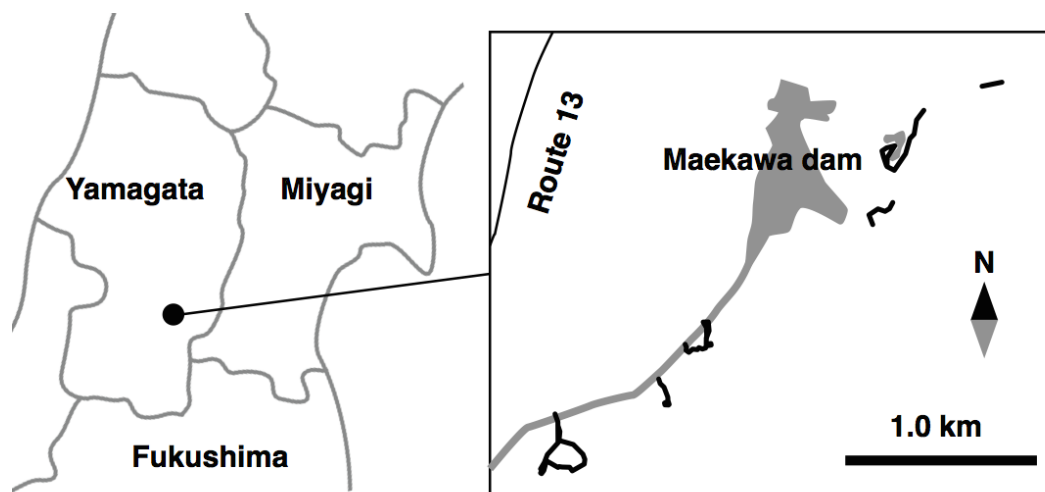

Supplement: S1 Fig — The trails are shown in thick lines. (PDF) [file pone.0143443.s001.pdf]

S2 Fig.

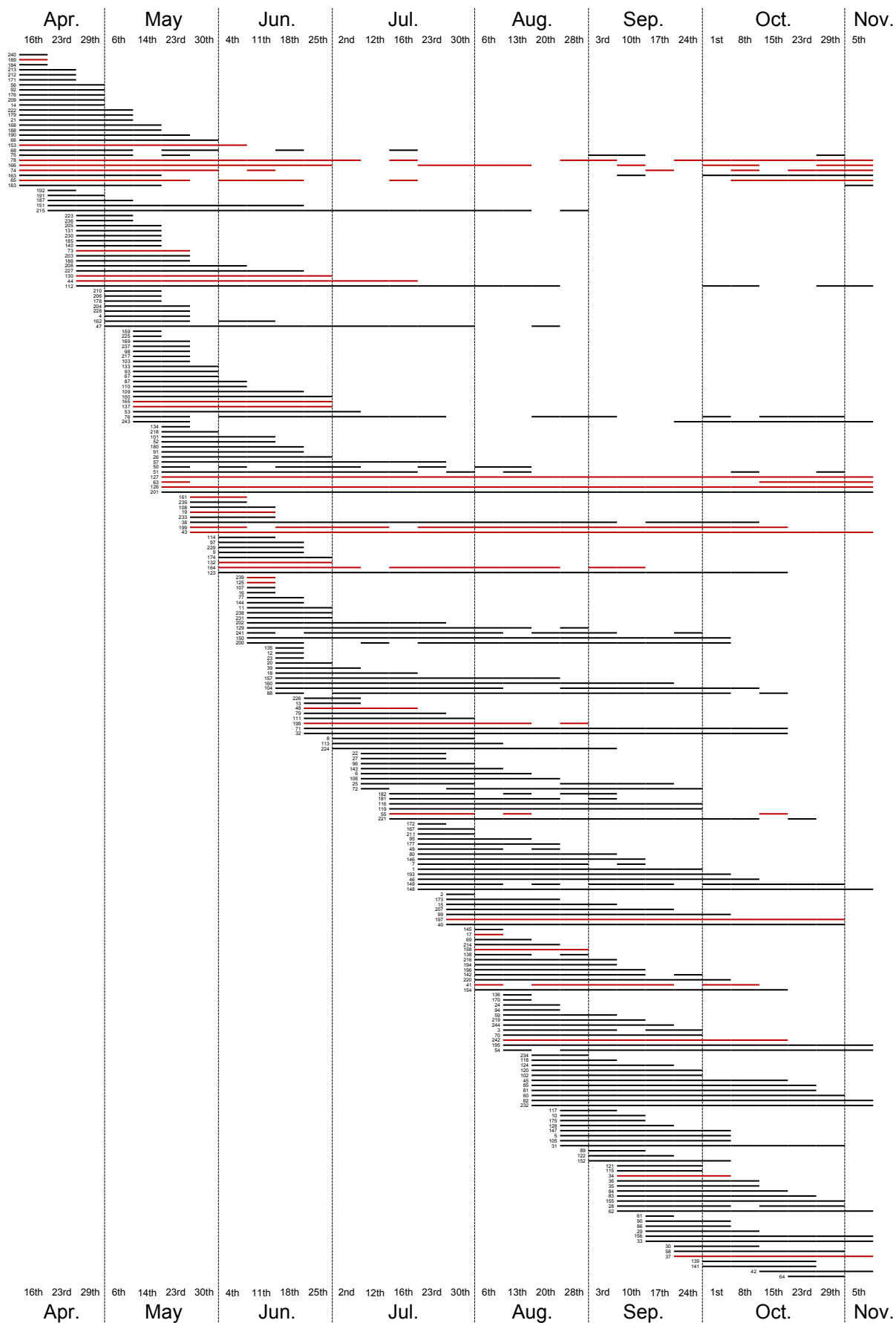

Supplement: S2 Fig — The horizontal line indicates weeks in which a species was seen flowering. Alien species are shown in red. The number on the left side of a line is a species ID (S1 Table). (PDF) [file pone.0143443.s002.pdf]

S3 Fig.

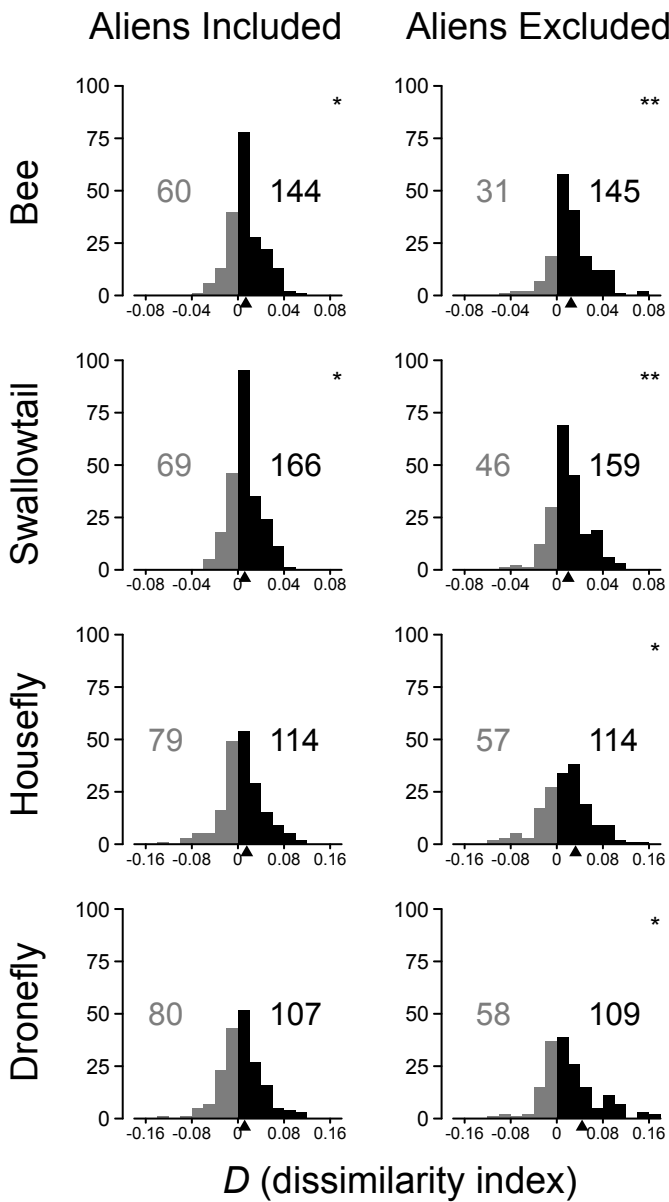

Supplement: S3 Fig — The left column shows the results for both native and alien species (n = 244); the right column shows the results excluding aliens (n = 212). In this analysis, similarly colored species in the same genus were grouped and treated as a single species (see S3 Table for the method and the list of grouped species). The number of species with negative D (left) and positive D (right) is shown in each panel. Unlike Fig 1, the numbers of natives are not shown on the left panels because some groups contain both natives and aliens. The triangle below the x-axis indicates the mean D. The symbols show the results of randomization tests for the mean D (*, P < 0.05; **, P < 0.01). (PDF) [file pone.0143443.s003.pdf]
