# Supplementary material for: Nonrandom Composition of Flower Colors in a Plant Community: Mutually Different Co-Flowering Natives and Disturbance by Aliens
Source: PLoS One. 2015 Dec 9;10(12):e0143443. doi: 10.1371/journal.pone.0143443 (PMC4674055; doi:10.1371/journal.pone.0143443)
Supplement: S1 Table — (PDF) [file pone.0143443.s004.pdf]

S1 Table. Species List

ID	Order	Family	Scientific name	Origin	Measured location	*n*
1	Alismatales	Alismataceae	*Alisma canaliculatum* A.Braun et C.D.Bouché	native	petal	10
2			*Sagittaria aginashi* Makino	native	petal	10
3			*Sagittaria trifolia* L.	native	petal	10
4		Araceae	*Arisaema peninsulae* Nakai	native	spathe	10
5	Apiales	Apiaceae	*Angelica decursiva* (Miq.) Franch. et Sav.	native	petal	10
6			*Cryptotaenia canadensis* (L.) DC. subsp. *japonica* (Hassk.) Hand.-Mazz.	native	petal	10
7			*Oenanthe javanica* (Blume) DC. subsp. *rosthornii* (Diels) F.T.Pu	native	petal	10
8			*Sanicula chinensis* Bunge	native	petal	10
9			*Torilis scabra* (Thunb.) DC.	native	petal	10
10		Araliaceae	*Aralia elata* (Miq.) Seem.	native	petal	10
11	Aquifoliales	Aquifoliaceae	*Ilex crenata* Thunb. var. *radicans* (Nakai) Murai	native	petal	10
12			*Ilex nipponica* Makino	native	petal	10
13	Asparagales	Amaryllidaceae	*Allium macrostemon* Bunge	native	petal	10
14			*Allium monanthum* Maxim.	native	petal	10
15		Asparagaceae	*Hosta sieboldii* (Paxton) J.W.Ingram var. *sieboldii* f. *spathulata* (Miq.) W.G.Schmid	native	petal	10
16			*Polygonatum lasianthum* Maxim.	native	petal (outer region)	10
17		Iridaceae	*Crocosmia* x *crocosmiiflora* (Lemoine) N.E.Br.	alien	petal (outer region)	10
18			*Iris ensata* Thunb. var. *spontanea* (Makino) Nakai ex Makino et Nemoto	native	petal (outer region)	10
19			*Iris pseudacorus* L.	alien	petal (outer region)	10
20		Orchidaceae	*Cremastra appendiculata* (D.Don) Makino var. *variabilis* (Blume) I.D.Lund	native	sepal	10
21			*Cymbidium goeringii* (Rchb.f.) Rchb.f.	native	sepal (outer region)	10
22			*Epipactis thunbergii* A.Gray	native	tepal	10
23			*Gastrodia elata* Blume	native	petal	10
24			*Goodyera schlechtendaliana* Rchb.f.	native	tepal	10
25			*Spiranthes sinensis* (Pers.) Ames var. *amoena* (M.Bieb.) H.Hara	native	tepal	10
26		Xanthorrhoeaceae	*Hemerocallis dumortieri* C.Morren var. *esculenta* (Koidz.) Kitam. ex M.Matsuoka et M.Hotta	native	petal (outer region)	10
27			*Hemerocallis fulva* L. var. *kwanso* Regel	native	petal (outer region)	10
28	Asterales	Asteraceae	*Adenocaulon himalaicum* Edgew.	native	petal	10
29			*Ainsliaea acerifolia* Sch.Bip. var. *subapoda* Nakai	native	ray	10
30			*Artemisia keiskeana* Miq.	native	floret	10
31			*Aster glehnii* F.Schmidt var. *hondoensis* Kitam.	native	ray	10
32			*Aster iinumae* Kitam.	native	ray	10
33			*Aster microcephalus* (Miq.) Franch. et Sav. var. *ovatus* (Franch. et Sav.) Soejima et Mot.Ito	native	ray	10
34			*Aster novi-belgii* L.	alien	ray	10
35			*Aster scaber* Thunb.	native	ray	10
36			*Atractylodes ovata* (Thunb.) DC.	native	petal	10
37			*Bidens frondosa* L.	alien	ray	10
38			*Cirsium japonicum* Fisch. ex DC.	native	floret	10
39			*Cirsium nipponense* (Nakai) Koidz.	native	floret	10
40			*Cirsium nipponicum* (Maxim.) Makino var. *nipponicum*	native	floret	10

S1 Table. Species List (Continued)

ID	Order	Family	Scientific name	Origin	Measured location	*n*
41	Asterales (cont.)	Asteraceae (cont.)	*Erigeron canadensis* L.	alien	floret	10
42			*Eclipta thermalis* Bunge	native	floret	10
43			*Erigeron annuus* (L.) Pers	alien	ray	10
44			*Erigeron philadelphicus* L.	alien	ray	10
45			*Eupatorium lindleyanum* DC. var. *lindleyanum*	native	floret	10
46			*Eupatorium makinoi* T.Kawahara et Yahara	native	floret	10
47			*Pseudognaphalium affine* (D.Don) Anderb	native	floret	10
48			*Hypochaeris radicata* L.	alien	ray (outer region)	10
49			*Inula salicina* L. var. *asiatica* Kitam.	native	ray (outer region)	10
50			*Ixeridium dentatum* (Thunb.) Tzvelev subsp. *dentatum*	native	ray (outer region)	10
51			*Ixeridium dentatum* (Thunb.) Tzvelev subsp. *nipponicum* (Nakai) J.H.Pak et Kawano var. *albiflorum* (Makino) Tzvelev f. *amplifolium* (Kitam.) H.Nakai et H.Ohashi	native	ray (outer region)	10
52			*Ixeris japonica* (Burm.f.) Nakai	native	ray (outer region)	10
53			*Ixeris stolonifera* A.Gray	native	ray (outer region)	10
54			*Lactuca indica* L.	native	ray	10
55			*Leontodon taraxacoides* (Vill.) Mérat	alien	ray (outer region)	10
56			*Petasites japonicus* (Siebold et Zucc.) Maxim.	native	floret (both sexes)	20
57			*Picris hieracioides* L. subsp. *japonica* (Thunb.) Krylov	native	ray (outer region)	10
58			*Prenanthes tanakae* (Franch. et Sav. ex Y.Tanaka et Ono) Koidz.	native	ray (outer region)	10
59			*Senecio cannabifolius* Less.	native	ray (outer region)	10
60			*Serratula coronata* L. subsp. *insularis* (Iljin) Kitam	native	floret	10
61			*Sigesbeckia pubescens* (Makino) Makino	native	ray	10
62			*Solidago virgaurea* L. Subsp. *Asiatica* (Nakai ex H.Hara) Kitam. Ex H.Hara	native	ray	10
63			*Sonchus asper* (L.) Hill	alien	ray	10
64			*Synurus pungens* (Franch. et Sav.) Kitam.	native	floret	10
65			*Taraxacum officinale* Weber ex F.H.Wigg.	alien	ray (outer region)	10
66			*Taraxacum venustum* H.Koidz.	native	ray (outer region)	10
67			*Tephroseris pierotii* (Miq.) Holub	native	ray	10
68			*Youngia japonica* (L.) DC.	native	ray (outer region)	10
69		Campanulaceae	*Adenophora divaricata* Franch. et Sav.	native	petal	10
70			*Adenophora triphylla* (Thunb.) A.DC. var. *japonica* (Regel) H.Hara	native	petal	10
71			*Lobelia chinensis* Lour.	native	petal (outer region)	10
72			*Platycodon grandiflorus* (Jacq.) A.DC.	native	petal (outer region)	10
73	Brassicales	Brassicaceae	*Barbarea vulgaris* R.Br.	alien	petal (outer region)	10
74			*Cardamine hirsuta* L.	alien	petal	10
75			*Cardamine scutata* Thunb.	native	petal	10
76			*Rorippa palustris* (L.) Besser	native	petal	10
77	Caryophyllales	Caryophyllaceae	*Sagina japonica* (Sw.) Ohwi	native	petal	10
78			*Stellaria media* (L.) Vill.	alien	petal	10
79		Droseraceae	*Drosera rotundifolia* L.	native	petal	10
80		Polygonaceae	*Fallopia japonica* (Houtt.) Ronse Decr. var. *japonica*	native	petal	10

S1 Table. Species List (Continued)

ID	Order	Family	Scientific name	Origin	Measured location	*n*
81	Caryophyllales (cont.)	Polygonaceae (cont.)	*Persicaria filiformis* (Thunb.) Nakai ex W.T.Lee	native	sepal	10
82			*Persicaria longiseta* (Bruijn) Kitag.	native	sepal	10
83			*Persicaria posumbu* (Buch.-Ham. ex D.Don) H.Gross	native	sepal	10
84			*Persicaria sagittata* (L.) H.Gross	native	sepal	10
85			*Persicaria thunbergii* (Siebold et Zucc.) H.Gross	native	sepal (outer region)	10
86		Portulacaceae	*Portulaca oleracea* L.	native	petal	10
87	Celastrales	Celastraceae	*Euonymus alatus* (Thunb.) Siebold f. *alatus*	native	petal	10
88	Commelinales	Commelinaceae	*Commelina communis* L.	native	petal	10
89			*Murdannia keisak* (Hassk.) Hand.-Mazz.	native	petal	7
90		Pontederiaceae	*Monochoria vaginalis* (Burm.f.) C.Presl ex Kunth	native	petal	10
91	Cornales	Cornaceae	*Cornus kousa* Buerger ex Hance subsp. *kousa*	native	bract	10
92	Crossosomatales	Stachyuraceae	*Stachyurus praecox* Siebold et Zucc.	native	petal (both sexes)	20
93			*Staphylea bumalda* DC.	native	petal	10
94	Dioscoreales	Dioscoreaceae	*Dioscorea japonica* Thunb.	native	male petal	10
95			*Dioscorea tokoro* Makino	native	petal (both sexes)	20
96		Nartheciaceae	*Aletris luteoviridis* (Maxim.) Franch.	native	petal	10
97	Dipsacales	Adoxaceae	*Viburnum dilatatum* Thunb.	native	petal	10
98			*Viburnum wrightii* Miq.	native	petal	10
99		Caprifoliaceae	*Patrinia scabiosifolia* Fisch. ex Trevir.	native	petal	10
100			*Valeriana flaccidissima* Maxim.	native	petal	10
101			*Weigela hortensis* (Siebold et Zucc.) K.Koch	native	petal (outer region)	10
102			*Dipsacus japonicus* Miq.	native	floret (distal region)	10
103			*Abelia spathulata* Siebold et Zucc. var. *spathulata*	native	petal (outer region)	10
104	Ericales	Balsaminaceae	*Impatiens noli-tangere* L.	native	petal (outer region)	10
105			*Impatiens textorii* Miq.	native	petal (outer region)	10
106		Clethraceae	*Clethra barbinervis* Siebold et Zucc.	native	petal	10
107		Ebenaceae	*Diospyros kaki* Thunb.	native	petal	10
108		Ericaceae	*Rhododendron multiflorum* (Maxim.) Craven	native	petal (outer region)	10
109			*Rhododendron kaempferi* Planch. var. *kaempferi*	native	petal (outer region)	10
110			*Rhododendron molle* (Blume) G.Don subsp. *japonicum* (A.Gray) K.Kron	native	petal (outer region)	10
111		Primulaceae	*Lysimachia clethroides* Duby	native	petal	10
112			*Lysimachia japonica* Thunb.	native	petal (outer region)	10
113			*Lysimachia vulgaris* L. var. *davurica* (Ledeb.) R.Knuth	native	petal (outer region)	10
114		Styracaceae	*Styrax japonica* Siebold et Zucc.	native	petal	10
115	Fabales	Fabaceae	*Amphicarpaea bracteata* (L.) Fernald subsp. *edgeworthii* (Benth.) H.Ohashi var. *japonica* (Oliv.) H.Ohashi	native	banner (outer region)	10
116			*Hylodesmum podocarpum* (DC.) H.Ohashi & R.R.Mill subsp. *oxyphyllum* (DC.) H.Ohashi & R.R.Mill var. *japonicum* (Miq.) H.Ohashi	native	banner (outer region)	10
117			*Glycine max* (L.) Merr. subsp. *soja* (Siebold et Zucc.) H.Ohashi	native	banner	10
118			*Kummerowia striata* (Thunb.) Schindl.	native	banner	10
119			*Lespedeza bicolor* Turcz.	native	banner (outer region)	10
120			*Lespedeza cuneata* (Dum.Cours.) G.Don var. *serpens* (Nakai) Ohwi ex Shimabuku	native	banner (outer region)	10

S1 Table. Species List (Continued)

ID	Order	Family	Scientific name	Origin	Measured location	*n*
121	Fabales (cont.)	Fabaceae (cont.)	*Lespedeza cuneata* (Dum.Cours.) G.Don	native	banner (outer region)	10
122			*Lespedeza pilosa (Thunb.)* Siebold et Zucc.	native	banner (outer region)	10
123			*Lotus corniculatus* L. var. *japonicus* Regel	native	banner	10
124			*Pueraria lobata* (Willd.) Ohwi	native	banner (outer region)	10
125			*Trifolium hybridum* L.	alien	newer banner	10
126			*Trifolium pratense* L.	alien	banner	10
127			*Trifolium repens* L.	alien	banner	10
128			*Vicia nipponica* Matsum.	native	banner (outer region)	10
129			*Vicia pseudo-orobus* Fisch. et C.A.Mey.	native	banner	10
130			*Vicia sativa* L. subsp. *nigra* (L.) Ehrh.	alien	banner (outer region)	10
131			*Vicia tetrasperma* (L.) Schreb.	native	banner	10
132			*Vicia villosa* Roth subsp. *varia* (Host) Corb.	alien	banner	10
133			*Wisteria floribunda* (Willd.) DC.	native	banner (outer region)	10
134		Polygalaceae	*Polygala japonica* Houtt.	native	petal	10
135	Fagales	Fagaceae	*Castanea crenata* Siebold et Zucc.	native	male petal	10
136	Gentianales	Apocynaceae	*Metaplexis japonica* (Thunb.) Makino	native	newer petal	10
137			*Vinca major* L.	alien	petal	10
138			*Vincetoxicum sublanceolatum* (Miq.) Maxim. var. *macranthum* Maxim.	native	petal	10
139		Gentianaceae	*Gentiana scabra* Bunge var. *buergeri* (Miq.) Maxim. ex Franch. et Sav.	native	petal (outer region)	10
140			*Gentiana zollingeri* Fawc.	native	petal (outer region)	10
141			*Swertia japonica* (Schult.) Makino	native	petal	10
142			*Tripterospermum japonicum* (Siebold et Zucc.) Maxim.	native	petal	10
143		Rubiaceae	*Galium trifidum* L. subsp. *columbianum* (Rydb.) Hultén	native	petal	10
144			*Mitchella undulata* Siebold et Zucc	native	petal	10
145			*Neanotis hirsuta* (L.f.) W.H.Lewis var. *hirsuta*	native	petal	10
146			*Paederia foetida* L.	native	petal (outer region)	10
147			*Rubia argyi* (H.Lév. et Vaniot) H.Hara ex Lauener et D.K.Ferguson	native	petal	10
148	Geraniales	Geraniaceae	*Geranium thunbergii* Siebold ex Lindl. et Paxton	native	petal	10
149	Lamiales	Lamiaceae	*Clinopodium chinense* (Benth.) Kuntze subsp. *grandiflorum* (Maxim.) H.Hara	native	petal	10
150			*Clinopodium micranthum* (Regel) H.Hara var. *micranthum*	native	petal	10
151			*Glechoma hederacea* L. subsp. *grandis* (A.Gray) H.Hara	native	lower lip (outer region)	10
152			*Isodon inflexus* (Thunb.) Kudô	native	petal	10
153			*Lamium purpureum* L.	alien	petal	10
154			*Lycopus maackianus* (Maxim. ex Herder) Makino	native	petal	10
155			*Mosla dianthera* (Buch.-Ham. ex Roxb.) Maxim.	native	petal	10
156			*Mosla scabra* (Thunb.) C.Y.Wu et H.W.Li	native	petal	10
157			*Prunella vulgaris* L. subsp. *asiatica* (Nakai) H.Hara	native	upper lip	10
158		Linderniaceae	*Lindernia dubia* (L.) Pennell subsp. *major* (Pursh) Pennell	alien	petal	10
159		Oleaceae	*Fraxinus sieboldiana* Blume	native	petal	10
160		Orobanchaceae	*Melampyrum roseum* Maxim. var. *japonicum* Franch. et Sav.	native	lower lip (outer region)	10

S1 Table. Species List (Continued)

ID	Order	Family	Scientific name	Origin	Measured location	*n*
161	Lamiales (cont.)	Paulowniaceae	*Paulownia tomentosa* (Thunb.) Steud.	alien	upper lip	10
162		Phrymaceae	*Mazus miquelii* Makino	native	lower lip (outer region)	10
163			*Mazus pumilus* (Burm.f.) Steenis	native	lower lip (outer region)	10
164		Plantaginaceae	*Plantago lanceolata* L	alien	anther	10
165			*Veronica arvensis* L.	alien	petal	10
166			*Veronica persica* Poir.	alien	petal (outer region)	10
167			*Veronicastrum japonicum* (Nakai) T.Yamaz. var. *japonicum*	native	petal	10
168	Laurales	Lauraceae	*Lindera umbellata* Thunb.	native	petal	10
169	Liliales	Colchicaceae	*Disporum smilacinum* A.Gray	native	petal (outer region)	10
170		Liliaceae	*Cardiocrinum cordatum* (Thunb.) Makino	native	petal (outer region)	10
171			*Gagea lutea* (L.) Ker Gawl.	native	petal (outer region)	10
172			*Lilium auratum* Lindl.	native	petal (white area)	10
173			*Lilium leichtlinii* Hook.f. f. *pseudotigrinum* (Carrière) H.Hara et Kitam.	native	petal (orange area)	10
174			*Lilium rubellum* Baker	native	petal (outer region)	10
175			*Tricyrtis affinis* Makino	native	petal (white area)	10
176		Melanthiaceae	*Helonias orientalis* (Thunb.) N.Tanaka	native	petal	10
177			*Veratrum maackii* Regel var. *parviflorum* (Maxim. ex Miq.) H.Hara	native	petal	10
178		Smilacaceae	*Smilax china* L.	native	petal	10
179	Magnoliales	Magnoliaceae	*Magnolia salicifolia* (Siebld et Zucc.) Maxim.	native	petal	10
180	Malpighiales	Euphorbiaceae	*Euphorbia lasiocaula* Boiss.	native	petal	10
181		Hypericaceae	*Hypericum erectum* Thunb.	native	petal (outer region)	10
182			*Hypericum laxum* (Blume) Koidz.	native	petal	10
183		Violaceae	*Viola grypoceras* A.Gray var. *grypoceras*	native	petal (outer region)	10
184			*Viola hondoensis* W.Becker et H.Boissieu	native	petal (outer region)	4
185			*Viola kusanoana* Makino	native	petal (outer region)	10
186			*Viola mandshurica* W.Becker	native	petal (outer region)	10
187			*Viola obtusa* Makino	native	petal (outer region)	10
188			*Viola rostrata* Pursh	native	petal (outer region)	10
189			*Viola sororia* Willd.	alien	petal (outer region)	10
190			*Viola verecunda* A.Gray	native	upper petal	10
191			*Viola violacea* Makino var. *makinoi* (H.Boissieu) Hiyama ex F.Maek.	native	petal (outer region)	10
192			*Viola yedoensis* Makino	native	petal (outer region)	7
193	Myrtales	Lythraceae	*Lythrum anceps* (Koehne) Makino	native	petal	10
194		Onagraceae	*Circaea mollis* Siebold et Zucc.	native	petal	10
195			*Epilobium pyrricholophum* Franch. et Sav.	native	petal	10
196			*Ludwigia epilobioides* Maxim.	native	petal	10
197			*Oenothera biennis* L.	alien	petal (outer region)	10
198			*Oenothera glazioviana* Micheli	alien	petal (outer region)	10
199	Nymphaeales	Nymphaeaceae	*Nymphaea* sp.	alien	petal	10
200			*Nymphaea tetragona* Georgi	native	petal	10

S1 Table. Species List (Continued)

ID	Order	Family	Scientific name	Origin	Measured location	*n*
201	Oxalidales	Oxalidaceae	*Oxalis* sp.	native	petal (outer region)	20
202	Piperales	Saururaceae	*Houttuynia cordata* Thunb.	native	bract	10
203	Ranunculales	Berberidaceae	*Epimedium koreanum* Nakai	native	petal	10
204		Lardizabalaceae	*Akebia quinata* (Houtt.) Decne.	native	female petal	10
205			*Akebia trifoliata* (Thunb.) Koidz.	native	female petal	10
206			*Akebia* x *pentaphylla* (Makino) Makino	native	female petal	10
207		Menispermaceae	*Cocculus trilobus* (Thunb.) DC.	native	petal	10
208		Papaveraceae	*Corydalis incisa* (Thunb.) Pers.	native	lower lip	10
209			*Corydalis leariloba* Siebold et Zucc.	native	lower lip	10
210			*Corydalis pallida* (Thunb.) Pers. var. *tenuis* Yatabe	native	lower lip	10
211			*Macleaya cordata* (Willd.) R.Br.	native	sepal	10
212		Ranunculaceae	*Anemone pseudoaltaica* H.Hara	native	petal	8
213			*Anemone raddeana* Regel	native	petal	10
214			*Clematis apiifolia* DC. var. *apiifolia*	native	sepal	10
215			*Ranunculus cantoniensis* DC.	native	petal	10
216			*Thalictrum minus* L. var. *hypoleucum* (Siebold et Zucc.) Miq.	native	sepal	10
217	Rosales	Elaeagnaceae	*Elaeagnus multiflora* Thunb.	native	young petal	10
218			*Elaeagnus umbellata* Thunb. var. *umbellata*	native	young petal	10
219		Rhamnaceae	*Berchemia racemosa* Siebold et Zucc.	native	petal	10
220		Rosaceae	*Agrimonia nipponica* Koidz.	native	petal	10
221			*Agrimonia pilosa* Ledeb. var. *japonica* (Miq.) Nakai	native	petal	10
222			*Cerasus apetala* (Siebold et Zucc.) Ohle ex H.Ohba var. *tetsuyae* H.Ohba	native	petal	10
223			*Cerasus leveilleana* (Koehne) H.Ohba	native	petal	10
224			*Geum japonicum* Thunb.	native	petal (outer region)	10
225			*Malus toringo* (Siebold) Siebold ex de Vriese	native	petal	10
226			*Potentilla centigrana* Maxim.	native	petal	10
227			*Potentilla freyniana* Bornm.	native	petal (outer region)	10
228			*Potentilla fragarioides* L. var. *major* Maxim.	native	petal (outer region)	10
229			*Rosa multiflora* Thunb.	native	petal	10
230			*Rubus palmatus* Thunb. var. *coptophyllus* (A.Gray) Kuntze ex Koidz.	native	petal	10
231			*Rubus parvifolius* L.	native	newer petal	10
232			*Sanguisorba tenuifolia* Fisch. ex Link	native	petal	10
233			*Neillia incisa* (Thunb.) S.H.Oh	native	petal	10
234		Urticaceae	*Laportea bulbifera* (Siebold et Zucc.) Wedd.	native	petal	10
235	Sapindales	Sapindaceae	*Acer ginnala* Maxim. var. *aidzuense* (Franch.) Pax	native	petal	10
236			*Acer japonicum* Thunb.	native	petal	10
237			*Acer pictum* Thunb.	native	petal	10
238	Saxifragales	Crassulaceae	*Sedum bulbiferum* Makino	native	petal	10
239			*Sedum sarmentosum* Bunge	alien	petal (outer region)	10
240		Hamamelidaceae	*Hamamelis japonica* Siebold et Zucc.	native	petal	10

S1 Table. Species List (Continued)

ID	Order	Family	Scientific name	Origin	Measured location	*n*
241	Solanales	Convolvulaceae	*Calystegia pubescens* Lindl.	native	petal (outer region)	10
242			*Cuscuta campestris* Yuncker	alien	petal	10
243	Undetermined	Boraginaceae	*Trigonotis peduncularis* (Trevir.) F.B.Forbes et Hemsl.	native	petal	10
244	Vitales	Vitaceae	*Ampelopsis glandulosa* (Wall.) Momiy. var. *heterophylla* (Thunb.) Momiy.	native	petal	10
