# Supplementary material for: Nonrandom Composition of Flower Colors in a Plant Community: Mutually Different Co-Flowering Natives and Disturbance by Aliens
Source: PLoS One. 2015 Dec 9;10(12):e0143443. doi: 10.1371/journal.pone.0143443 (PMC4674055; doi:10.1371/journal.pone.0143443)
Supplement: S2 Table — (PDF) [file pone.0143443.s005.pdf]

Table S2. Kendall's τ for correlations between E and flowering duration

Pollinator	Aliens included (n = 244)		Aliens excluded (n = 212)	
	τ	adjusted P	τ	adjusted P
Bee	0.0775	0.2208	0.1257	0.0727
Swallowtail	0.0650	0.2911	0.1066	0.1073
Housefly	0.0277	0.5863	0.0637	0.3375
Dronefly	0.0298	0.5863	0.0675	0.3375
