# Supplementary material for: Nonrandom Composition of Flower Colors in a Plant Community: Mutually Different Co-Flowering Natives and Disturbance by Aliens
Source: PLoS One. 2015 Dec 9;10(12):e0143443. doi: 10.1371/journal.pone.0143443 (PMC4674055; doi:10.1371/journal.pone.0143443)
Supplement: S3 Table — (PDF) [file pone.0143443.s006.pdf]

S3 Table. List of similarly colored species in the same genus for each pollinator vision

Genus	Bee		Swallowtail		Housefly		Dronefly	
	Included	Excluded	Included	Excluded	Included	Excluded	Included	Excluded
Acer	(235, 236)	(235, 236)			(236, 237)	(236, 237)	(236, 237)	(236, 237)
Adenophora	(69, 70)	(69, 70)			(69, 70)	(69, 70)	(69, 70)	(69, 70)
Agrimonia	(220, 221)	(220, 221)	(220, 221)	(220, 221)	(220, 221)	(220, 221)	(220, 221)	(220, 221)
Akebia	(204, 206)	(204, 206)			(204, 205, 206)	(204, 205, 206)		
Allium					(13, 14)	(13, 14)	(13, 14)	(13, 14)
Aster	(31, 32, 33, 35)	(31, 32, 33, 35)	(32, 33, 35)	(32, 33, 35)	(32, 33, 34, 35)	(32, 33, 35)	(31, 32, 33, 34, 35)	(31, 32, 33, 35)
Cardamine	(74, 75)				(74, 75)		(74, 75)	
Cerasus	(222, 223)	(222, 223)	(222, 223)	(222, 223)	(222, 223)	(222, 223)	(222, 223)	(222, 223)
Cirsium	(38, 39, 40)	(38, 39, 40)			(38, 39, 40)	(38, 39, 40)	(38, 39, 40)	(38, 39, 40)
Clinopodium	(149, 150)	(149, 150)			(149, 150)	(149, 150)	(149, 150)	(149, 150)
Corydalis					(208, 210)	(208, 210)	(208, 210)	(208, 210)
Dioscorea					(94, 95)	(94, 95)		
Elaeagnus	(217, 218)	(217, 218)			(217, 218)	(217, 218)	(217, 218)	(217, 218)
Erigeron	(43, 44)				(43, 44)		(43, 44)	
Eupatorium	(45, 46)	(45, 46)			(45, 46)	(45, 46)	(45, 46)	(45, 46)
Gentiana	(139, 140)	(139, 140)			(139, 140)	(139, 140)	(139, 140)	(139, 140)
Hemerocallis	(26, 27)	(26, 27)					(26, 27)	(26, 27)
Hypericum	(181, 182)	(181, 182)	(181, 182)	(181, 182)	(181, 182)	(181, 182)	(181, 182)	(181, 182)
Ilex	(11, 12)	(11, 12)						
Ixeris	(51, 52)	(51, 52)	(51, 52)	(51, 52)	(51, 52)	(51, 52)	(51, 52, 53)	(51, 52, 53)
Lespedeza	(120, 121, 122)	(120, 121, 122)			(120, 121, 122)	(120, 121, 122)	(120, 122)	(120, 122)
Lilium	(172, 174)	(172, 174)			(172, 174)	(172, 174)	(172, 174)	(172, 174)
Mazus					(162, 163)	(162, 163)	(162, 163)	(162, 163)
Mosla	(155, 156)	(155, 156)			(155, 156)	(155, 156)	(155, 156)	(155, 156)
Nymphaea					(199, 200)		(199, 200)	
Oenothera							(197, 198)	
Persicaria	(82, 84, 85)	(82, 84, 85)	(82, 85)	(82, 85)	(81, 82, 84, 85)	(81, 82, 84, 85)	(81, 82, 83, 84, 85)	(81, 82, 83, 84, 85)
Potentilla	(227, 228)	(227, 228)			(226, 227, 228)	(226, 227, 228)	(226, 227, 228)	(226, 227, 228)
Rhododendron	(109, 110)	(109, 110)					(109, 110)	(109, 110)
Rubus							(230, 231)	(230, 231)
Sagittaria	(2, 3)	(2, 3)			(2, 3)	(2, 3)	(2, 3)	(2, 3)
Sedum					(238, 239)		(238, 239)	
Taraxacum					(65, 66)		(65, 66)	
Trifolium	(125, 127)		(125, 127)		(125, 127)		(125, 126, 127)	
Veronica	(165, 166)				(165, 166)		(165, 166)	
Viburnum	(97, 98)	(97, 98)			(97, 98)	(97, 98)	(97, 98)	(97, 98)
Vicia	(130, 131), (128, 129)	(128, 129)	(130, 131)		(128, 129, 130, 131, 132)	(128, 129, 131)	(128, 129, 130, 131, 132)	(128, 129, 131)
Viola	(184, 185, 192), (186, 187), (183, 188, 190, 191)	(184, 185, 192), (186, 187), (183, 188, 190, 191)			(183, 184, 185, 186, 187, 188, 189, 190, 191, 192)	(183, 184, 185, 186, 187, 188, 191, 192)	(183, 184, 185, 186, 187, 188, 189, 190, 191, 192)	(183, 184, 185, 186, 187, 188, 190, 191, 192)

The numbers are species IDs from S1 Table. Species in the same brackets were analyzed as a single species in S4 Table and S3 Fig.

For houseflies and droneflies, species in the same genus in the same color category were grouped.

For bees and swallowtails, at each step of grouping, the nearest points (species) in the color space were combined and the midpoint was computed for a new cluster. We repeated this step until the minimum distance between any two species in a genus exceeded 0.09 and 0.03 for bees and swallowtails, respectively. These values are the criteria for the recognition of a color difference (the same as those adopted in Ohashi et al. 2015). After grouping, the sample size (n) was reduced to the sum of the number of groups and uncombined species: e.g., for the analysis of both native and alien species with respect to bee vision, n was reduced from 244 to 204 (32 groups and 172 uncombined species).

The flowering period of each group was produced by merging the flowering periods of the group members.
