# Supplementary material for: Nonrandom Composition of Flower Colors in a Plant Community: Mutually Different Co-Flowering Natives and Disturbance by Aliens
Source: PLoS One. 2015 Dec 9;10(12):e0143443. doi: 10.1371/journal.pone.0143443 (PMC4674055; doi:10.1371/journal.pone.0143443)
Supplement: S4 Table — (PDF) [file pone.0143443.s007.pdf]

S4 Table. Mean D and D/E and the results of randomization tests for mean D after grouping similarly-colored species in the same genus

Pollinator	Aliens included			Aliens excluded		
	n	mean D (D/E)	adjusted P	n	mean D (D/E)	adjusted P
Bee	204	**0.0069** (0.0312)	0.0352	177	**0.0123** (0.0575)	0.0032
Swallowtail	235	**0.0059** (0.0283)	0.0456	205	**0.0099** (0.0477)	0.0072
Housefly	193	0.0077 (0.0116)	0.2053	172	**0.0163** (0.0266)	0.0197
Dronefly	188	0.0058 (0.0153)	0.4922	169	**0.0219** (0.0561)	0.0352

Significant Ds are in bold.
